# Impact of the changing landscape of induction therapy prior to autologous stem cell transplantation in 540 newly diagnosed myeloma patients: a retrospective real-world study

**DOI:** 10.1007/s00432-022-04184-x

**Published:** 2022-08-20

**Authors:** Song-Yau Wang, Tanja Holzhey, Simone Heyn, Thomas Zehrfeld, Susann Fricke, Franz Albert Hoffmann, Cornelia Becker, Leanthe Braunert, Thomas Edelmann, Inessa Paulenz, Marcus Hitzschke, Franziska Flade, Andreas Schwarzer, Klaus Fenchel, Georg-Nikolaus Franke, Vladan Vucinic, Madlen Jentzsch, Sebastian Schwind, Saskia Hell, Donata Backhaus, Thoralf Lange, Dietger Niederwieser, Markus Scholz, Uwe Platzbecker, Wolfram Pönisch

**Affiliations:** 1 Hematology and Cell Therapy, Medical Clinic and Policlinic 1, University Hospital Leipzig, University of Leipzig, Liebigstraße 22, 04103 Leipzig, Germany; 2 Hospital Torgau, Christianistraße 1, 04860 Torgau, Germany; 3 Hematology Practice, Biedermannstraße 84, 04277 Leipzig, Germany; 4 Hematology Practice, Theodor - Heuss-Str. 2, 04435 Schkeuditz, Germany; 5 Hospital Dessau, Auenweg 38, 06847 Dessau-Roßlau, Germany; 6 Hospital Borna, Rudolf-Virchow-Straße 2, 04552 Borna, Germany; 7 Hematology Practice, Strümpellstraße 42, 04289 Leipzig, Germany; 8 Hematology Practice, Bahnhofstraße 12, 07318 Saalfeld, Saale Germany; 9 Hospital Weißenfels, Naumburger Straße 76, 06667 Weißenfels, Germany; 10 Institute for Medical Informatics, Statistics and Epidemiology (IMISE), University of Leipzig, Härtelstraße 16‑18, 04107 Leipzig, Germany

**Keywords:** Multiple myeloma, Autologous stem cell transplantation, Bortezomib, Real-world evidence

## Abstract

**Introduction:**

Autologous stem cell transplantation (ASCT) is the standard treatment for younger patients with newly diagnosed multiple myeloma (MM). However, due to restrictive exclusion criteria, more than half of eligible patients are usually excluded from transplant studies.

**Methods:**

This retrospective monocentric analysis included 540 patients with MM who received an ASCT between 1996 and 2019.

**Results:**

Up to 2005, induction therapy consisted mainly of conventional chemotherapies, e.g. vincristine/doxorubicin/dexamethasone (VAD). In the following years, the triple-combinations based on bortezomib coupled with doxorubicin/dexamethasone (PAD), melphalan/prednisolone (VMP), cyclophposphamide/dexamethasone (VCD) or bendamustine/prednisolone (BPV) became the most popular treatment options. A progressive improvement in PFS was observed in patients treated with the two current induction therapies BPV (47 months) or VCD (54 months) compared to VAD (35 months, *p* < 0.03), PAD (39 months, *p* < 0.01 and VMP (36 months, *p* < 0.01). However, there was no significant difference in median OS (VAD 78, PAD 74, VMP 72, BPV 80 months and VCD not reached). In our analysis, we also included 139 patients who do fulfill at least one of the exclusion criteria for most phase 3 transplant studies (POEMS/amyloidosis/plasma cell leukemia, eGFR < 40 mL/min, severe cardiac dysfunction or poor general condition). Outcome for these patients was not significantly inferior compared to patients who met the inclusion criteria for most of the transplant studies with PFS of 36 vs 41 months (*p* = 0.78) and OS of 78 vs 79 months (*p* = 0.34).

**Conclusions:**

Our real-world data in unselected pts also stress the substantial value of ASCT during the first-line treatment of younger MM pts.

## Introduction

Multiple Myeloma (MM), the second most common hematologic malignancy, has an estimated incidence of more than nine cases per 100,000 with around 7600 new cases in Germany in 2020 (Robert Koch-Institut, 2020). The median age at MM diagnosis is 73 years with approximately 35% of patients being younger than 65 years (Klausen et al. 2019). For these patients, high-dose therapy followed by autologous stem cell transplantation (ASCT) is the standard treatment (Cavo et al. 2011). The Intergroupe Francophone du Myélome (IFM) 90 study was the first to demonstrate the superiority of ASCT over conventional chemotherapy (Attal et al. 1996). A meta-analysis examined nine randomized studies that compared conventional chemotherapy with high-dose chemotherapy and ASCT (Koreth et al. 2007). In most of these studies, ASCT significantly improved the rate of complete response (CR) and progression-free survival (PFS), although an overall survival (OS) benefit could only be demonstrated in three studies. Before the era of novel agents, the combination of vincristine, doxorubicin and dexamethasone (VAD) had long been the standard induction regimen prior to ASCT (Barlogie et al. 2006; Sonneveld et al. 2012). Trials published in the ASCT setting showed an overall response rate (ORR) ranging from 32 to 85% (CR 2–8%) after 2–4 VAD cycles and an ORR of 68–93% (CR 9–29%) after ASCT with a median PFS of 22–29 and OS of 47–70 months. In the last 15 years, the introduction of novel agents, particularly bortezomib, into induction therapy for transplant-eligible patients has markedly improved the management of MM. In the IFM 2005-01 study, bortezomib plus dexamethasone was shown to be a highly active induction treatment prior to ASCT, resulting in an ORR of 79% and a CR rate of 6% after induction therapy, and an ORR of 80% and a CR rate of 16% after subsequent ASCT, with a median event-free survival of 36 months and an OS of 81% at 3 years (Harousseau et al. 2010). Further intensification of the induction regimen to include three-drug combinations of bortezomib with alkylating chemotherapy (e.g. cyclophosphamide, bendamustine), anthracycline (doxorubicin) or immunomodulatory drugs (thalidomide and lenalidomide) has resulted in superior ORR and PFS. These triple combinations resulted in clinically relevant improvements in ORR (63–93%) and CR rate (7–35%) after induction therapy as well as increasing ORR (79–97%) and CR rates (21–44%) after ASCT with a median PFS of 35–55 months and a 3-year OS of 75–90% (Cavo et al. 2010; Moreau et al. 2011; Sonneveld et al. 2012; Sonneveld et al. 2013; Mai et al. 2015; Mateos et al. 2015; Attal et al. 2017; Tacchetti et al. 2020). These studies are also used to evaluate the efficacy and tolerability of the respective combination therapy and potentially resulting approvals. However, due to restrictive inclusion and exclusion criteria, more than half of patients with newly diagnosed MM (NDMM) are routinely excluded from randomized phase 3 trials with ASCT (Blimark et al. 2018; Klausen et al. 2019). In contrast, we included additional patients with kidney failure (estimated glomerular filtration rate (eGFR) < 40 mL/min), congestive heart failure (left ventricular ejection fraction < 40%), WHO performance status > 2 and other severe comorbidities in our study. With the aim of reflecting more closely on the conditions of routine practice in the changing treatment landscape, we conducted a retrospective study to determine the feasibility and efficacy for all NDMM patients treated in the Department of Hematology and Oncology at the University of Leipzig with a single, tandem ASCT or autologous/reduced-intensity conditioning allogeneic (auto-RICallo) SCT in the period from 1996 to 2019.

## Methods

### Patients

This retrospective analysis included all consecutive patients with NDMM who received first-line induction therapy followed by high-dose therapy and subsequent ASCT in the university hospital of Leipzig between January 1st, 1996 and December 31st, 2019. The data were collected from an electronic database containing patient records. All patients had a histologically or cytologically proven MM with symptomatic disease based on the CRAB-criteria of the International Myeloma Working Group (IMWG) (Rajkumar et al. 2014). Patients with significantly compromised general conditions were also considered. For better comparability, we have deployed the main exclusion criteria of the four-phase 3 studies [StaMINA trial (Stadtmauer et al. 2019); IFM2013-04 trial (Moreau et al. 2016); IFM2009 trial (Attal et al. 2017) and the EMN02/HO95 trial (Cavo et al. 2020)], which were also used by Klausen et al. (2019): POEMS/amyloidosis/plasma cell leukemia, eGFR < 40 mL/min, severe cardiac dysfunction (NYHA classification III-IV, ejection fraction < 40%), poor general condition caused mainly by MM with an Eastern Cooperative Oncology Group performance status (ECOG) 3/4, prior malignancies within 5 years or other severe comorbidities. All patients gave written informed consent for the applied treatment and the use of anonymized personal data for clinical research. This study was performed in line with the principles of the Declaration of Helsinki. Approval was granted by the Ethics Committee of the Medical Faculty, Leipzig University (IRB 00,001,750; registration number 118/18-e).

### Treatment protocols

The most commonly used induction treatments included VAD, bortezomib, melphalan and prednisone (VMP), bendamustine, prednisone and bortezomib (BPV), bortezomib and dexamethasone (VD), bortezomib, adriamycin and dexamethasone (PAD) and bortezomid, cyclophosphamide and dexamethasone (VCD).

Peripheral blood stem cell (PBSC) collection was performed 2–3 weeks after induction treatment. The mobilization regimen consisted of cyclophosphamide 4 g/m^2^ or in case of severe renal insufficiency or preexisting heart disease 2 g/m^2^. All patients received G-CSF (2 × 5 μg/kg) until the completion of stem cell collection. PBSC collection was started when the required number of CD 34^+^ cells (≥ 20 × 10^6^/L) was detected in peripheral blood. The target for all patients was to collect stem cells for two to three transplants. In patients with a poor stem cell yield in the first leukapheresis session, plerixafor was added before the next apheresis.

The pre-transplantation conditioning therapy consisted of melphalan 200 mg/m^2^. In case of concomitant heart amyloidosis or severe renal insufficiency, the dose of melphalan was reduced to 100 or 140 mg/m^2^. G-CSF (5 μg/kg body weight) was given on day 4 after stem cell reinfusion and continued until reconstitution of leukocytes ≥ 1.0 × 10^9^/L.

### Definition of response

Evaluation of response was based on the international uniform response criteria for multiple myeloma (Durie et al. 2006). In addition, the terms ‘near complete response’ (nCR) was included. Treatment responses were verified after the end of induction therapy and three months after the first ASCT. OS was measured from the start of induction treatment to the time of death, and PFS from the start of induction treatment to the time at which a relapse, progression or death was observed. The degree of improvement of renal function was assessed according to the criteria of the IMWG consensus statement (Dimopoulos et al. 2010).

### Evaluation of efficacy

Patients were examined within seven days prior to initiation of induction therapy. Staging was performed for each patient comprising medical history, physical examination including a detailed neurological examination, determination of World Health Organization Performance Status, determination of laboratory parameters (including β_2_-microglobulin, serum protein, serum protein electrophoresis, myeloma typing of serum and urine, serum-free light chain assay (Freelite®), serum creatinine, serum calcium and C-reactive protein), electrocardiogram, low dose CT and bone marrow examination. Myeloma protein concentration was determined by the integral of the area under the myeloma protein curve (based on electrophoresis data) and by relating it to the total serum protein. Renal function was assessed by the eGFR using the Modification of Diet in Renal Disease (MDRD) formula (Levey et al. 1999). Patients were followed-up at 3 to 4-weekly intervals during the period of induction therapy/ASCT and thereafter at 12-weekly intervals until disease progression.

### Statistical methods

Descriptive statistics were calculated for demographic and baseline variables. Regarding survival follow-up, the data set was freezed on May 15th, 2020. All patients who commenced treatment until this date were included in the analysis. OS and PFS were estimated by the Kaplan–Meier method. Survival curves are compared by Log Rank tests (IBM SPSS Statistics, Version 24). Transplant-related mortality (TRM) was determined as death from any cause other than progression or relapse before day + 100 from the first ASCT. *p*-values of group differences were calculated applying the Wilcoxon rank-sum test or Student’s t-test. Categorical variables were compared using the χ2-test. *p*-values < 0.05 were considered significant.

## Results

### Patient characteristics

This retrospective analysis included 540 patients with NDMM treated with induction therapy followed by ASCT in the first-line therapy. Baseline demographics and disease characteristics are shown in Table 1. Median age at diagnosis was 59 (range 29–75) years. There were 203 females (38%) and 337 males (62%).

**Table 1:** Patient characteristics of 540 newly diagnosed multiple myeloma patients grouped in four time-periods depending on the time of diagnosis

Parameter	Cohort 1 1996–2005 *n* = 71	Cohort 2 2006–2010 *n* = 125	Cohort 3 2011–2015 *n* = 191	Cohort 4 2016–2019 *n* = 153
Median age,	57	59	59	62
years (range)	(31–70)	(41–70)	(29–71)	(40–75)
*ECOG 0/1*, *n* (%)	45 (81)	85 (71)	98 (52)	97 (64)
≥ 2, *n* (%)	10 (19)	34 (29)	92 (48)	55 (36)
Unavailable, *n*	16	6	1	1
MM-Type				
IgG, * n* (%)	43 (61)	62 (50)	99 (52)	80 (52)
IgA, * n* (%)	11 (15)	28 (22)	37 (19)	36 (24)
IgD, * n* (%)	0	1 (1)	1 (1)	2 (2)
Light chain, *n* (%)	15 (21)	26 (21)	53 (28)	34 (22)
Asecretory, *n* (%)	2 (3)	8 (6)	1 (1)	1 (1)
ISS				
I, *n* (%)	24 (61)	66 (54)	94 (49)	78 (51)
II, *n* (%)	8 (21)	28 (23)	59 (31)	45 (29)
III, *n* (%)	7 (18)	28 (23)	38 (20)	30 (20)
Unavailable, *n*	32	3	0	0
Cytogenetics^a^				
High-risk^b^, *n* (%)	0	8 (13)	33 (23)	38 (32)
Standard-risk, *n* (%)	16 (100)	52 (87)	110 (77)	81 (68)
Unavailable, *n*	55	65	48	34
Transplant methods^c^				
Single ASCT, *n* (%)	40 (56)	62 (50)	158 (83)	134 (86)
Tandem ASCT, *n* (%)	6 (8)	49 (39)	12 (6)	15 (10)
Auto-RICallo-SCT, *n* (%)	25 (35)	14 (11)	21 (11)	4 (3)
Maintenance (IMiDs), *n* (%)	4 (6)	7 (6)	27 (14)	75 (49)
Median PFS (months)	39	36	39	43
Median OS (months)	80	78	74	nr

Abbreviation: nr not reached

^a^ Results available from 338 patients

^b^ High-risk: del(17p), *t*(4;14), *t*(14;16), *t*(16;20)

^c^Single ASCT: *n* = 394, tandem ASCT *n* = 82, auto-RICallo-SCT *n* = 64

### Response and survival

**Fig. 1:**
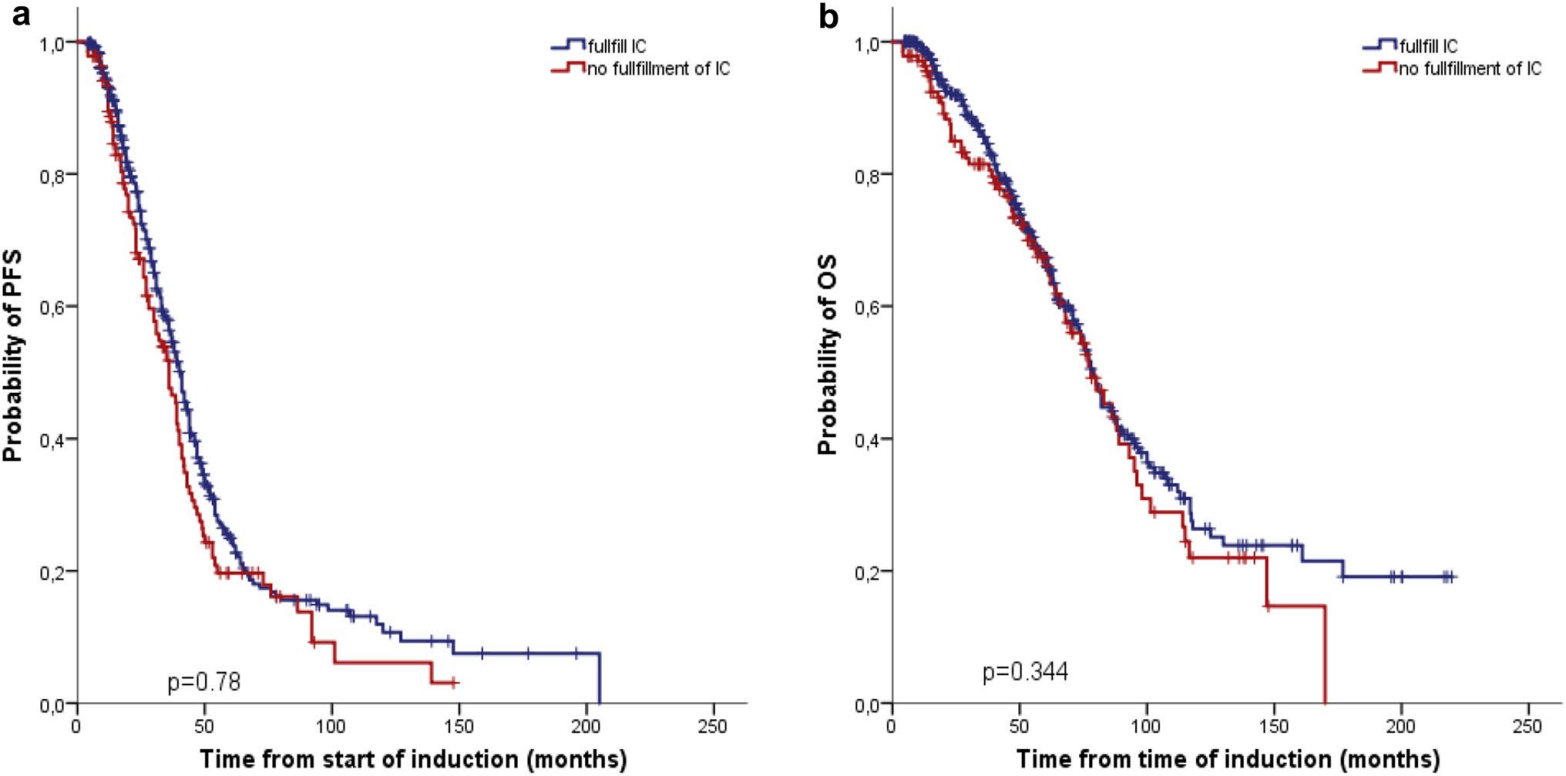
Progression-free survival (PFS) (**a**) and overall survival (OS) (**b**) of 139 patients who do not fulfill the most commonly used inclusion criteria of ASCT studies compared to 401 study-eligible patients. Outcome for those patients who did not meet the inclusion criteria was not significantly inferior to the outcome of those meeting the inclusion criteria for the majority of transplant studies: median PFS (36 vs 41 months; *p* = 0.78) and median OS (78 vs 79 months; *p* = 0.34)

The majority of patients (*n* = 430; 80%) responded after induction therapy with 21 stringent complete response (sCR) (4%), 11 CR (2%), 31 nCR (6%), 101 very good partial response (VGPR) (19%) and 266 partial response (PR) (49%). The median duration from the start of induction therapy to first ASCT was 159 (range 59–517) days. After the first ASCT, the ORR increased to 97% with 76 sCR (14%), 41 CR (8%), 88 nCR (16%), 168 VGPR (31%) and 149 PR (28%). TRM was 0.6% (*n* = 3), with two patients dying due to septicemia and one patient with intracerebral hemorrhage. With a median follow-up of 85 months, the median PFS was 39 (95% CI 36.7–41.3) and median OS 79 (95% CI 74.1–83.9) months. In accordance with the Danish MM registry (Klausen et al. 2019), we also included 139 (26%) patients in our analysis, who fulfilled at least one of the exclusion criteria for most clinical phase 3 transplant studies [POEMS/amyloidosis/plasma cell leukemia (*n* = 18, 3%), eGFR < 40 mL/min (*n* = 73, 14%), severe cardiac dysfunction (*n* = 15, 3%), ECOG 3/4 (*n* = 30, 6%), prior malignancies within 5 years (*n* = 21, 4%) and other severe comorbidities (*n* = 7, 1%)]. Outcome for these patients was not significantly inferior compared to those meeting the inclusion criteria for the majority of transplant studies: PFS of 36 vs 41 months (*p* = 0.78) and OS of 78 vs 79 months (*p* = 0.34) (Fig. 1 a, b). There was also no difference between the groups in terms of the ≥ CR rate (37/139, 27% vs 80/401, 20%, *p* = 0.10).

### Outcome according to the time of MM diagnosis

**Fig. 2:**
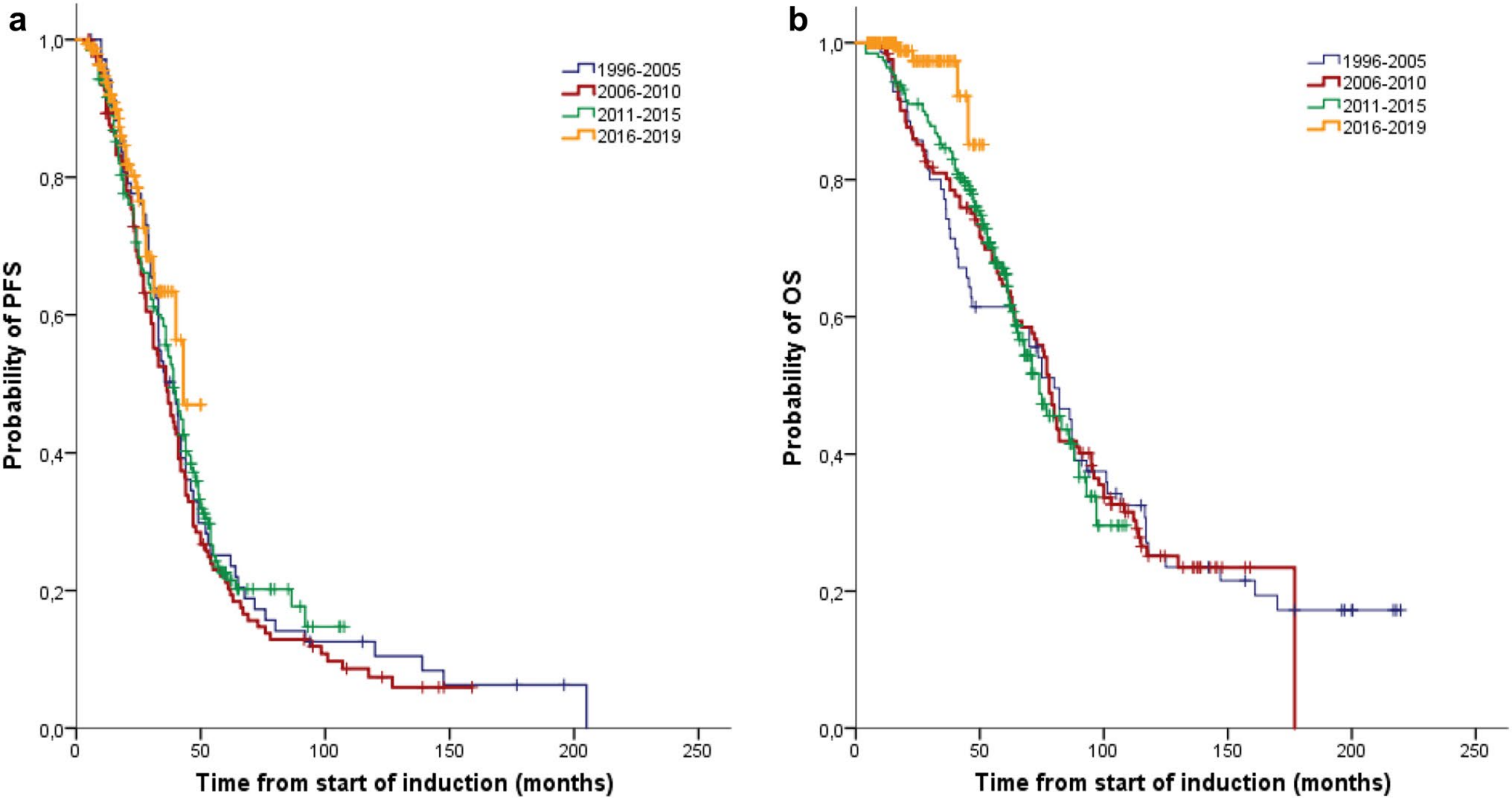
Progression-free survival (PFS) (**a**) and overall survival (OS) (**b**) according to time of MM diagnosis: 1996–2005 (*n* = 71), 2006–2010 (*n* = 125), 2011–2015 (*n* = 191) and 2016–2019 (*n* = 153). There was no significant difference in median PFS between the four cohorts (39 vs 36 vs 39 vs 43 months). While no improvement was seen in the median OS during the first three cohorts, the survival of the patients treated within the last four years was significantly better (*p* < 0.01)

According to the time of MM diagnosis, we divided the patients into four cohorts. Cohort 1 comprises 71 patients with their first MM diagnosis between 1996 and 2005, cohort 2 125 patients between 2006 and 2010, cohort 3 191 patients between 2011 and 2015 and cohort 4 153 patients between 2016 and 2019 (Table 1). The median age at diagnosis increased from 57 (range 31–70) years in the first cohort up to 62 (range 40–75) years in the period after 2015 (*p* < 0.001). There was no difference between the four cohorts regarding the subtype of MM and ISS stage. In the later period after 2010, we transplanted significantly more patients with a moderately/severe restricted general condition (ECOG ≥ 2) (*p* < 0.01). Between the first and last cohorts, there was a significant increase in both the ≥ VGPR rate (39 vs 81%, *p* < 0.001) and the ≥ CR rate (11 vs 25%, *p* < 0.03) after the first ASCT. However, there was no significant difference in PFS between the four cohorts (39 vs 36 vs 39 vs 43 months; *p* > *0.05*) (Fig. 2 a). While no improvement was seen in the OS during the first three cohorts, the survival of the patients treated within the last four years was significantly better (*p* < 0.01) (Fig. 2 b). The 48-months OS for patients diagnosed in the last cohort was 85% compared with 61% in cohort 1, 74% in cohort 2 and 76% in cohort 3. Based on EMA approval, 75 patients received lenalidomide maintenance therapy since 2017. With a short median follow-up time of only 17 months, there was no significant benefit in PFS and OS for the patients on maintenance therapy. No consolidation therapy was performed due to the lack of EU approval.

### Impact of induction therapies

**Fig. 3:**
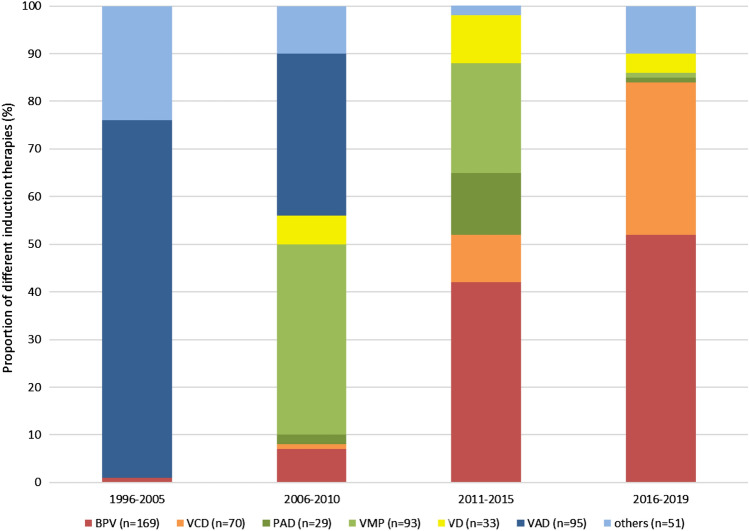
Changing landscape of induction therapies prior to autologous stem cell transplantation in 540 patients divided into four cohorts over time from 1996 to 2019. VAD: vincristine, adriamycin and dexamethasone; VMP: bortezomib, melphalan and prednisone; BPV: bendamustine, prednisone and bortezomib, VD: bortezomib and dexamethasone; PAD: bortezomb, adriamycin and dexamethasone; VCD: bortezomid, cyclophosphamide and dexamethasone. Others: Regimens used up to and including 2010: VCAP (vincristine, cyclophosphamide, adriblastin, prednisolone), BP (bendamustine and prednisolone) and from 2011: VTD (bortezomib, thalidomide and dexamethasone), VRD (bortezomid, lenalidomide and dexamethasone), DVD (daratumumab, bortezomib and dexamethasone)

**Fig. 4:**
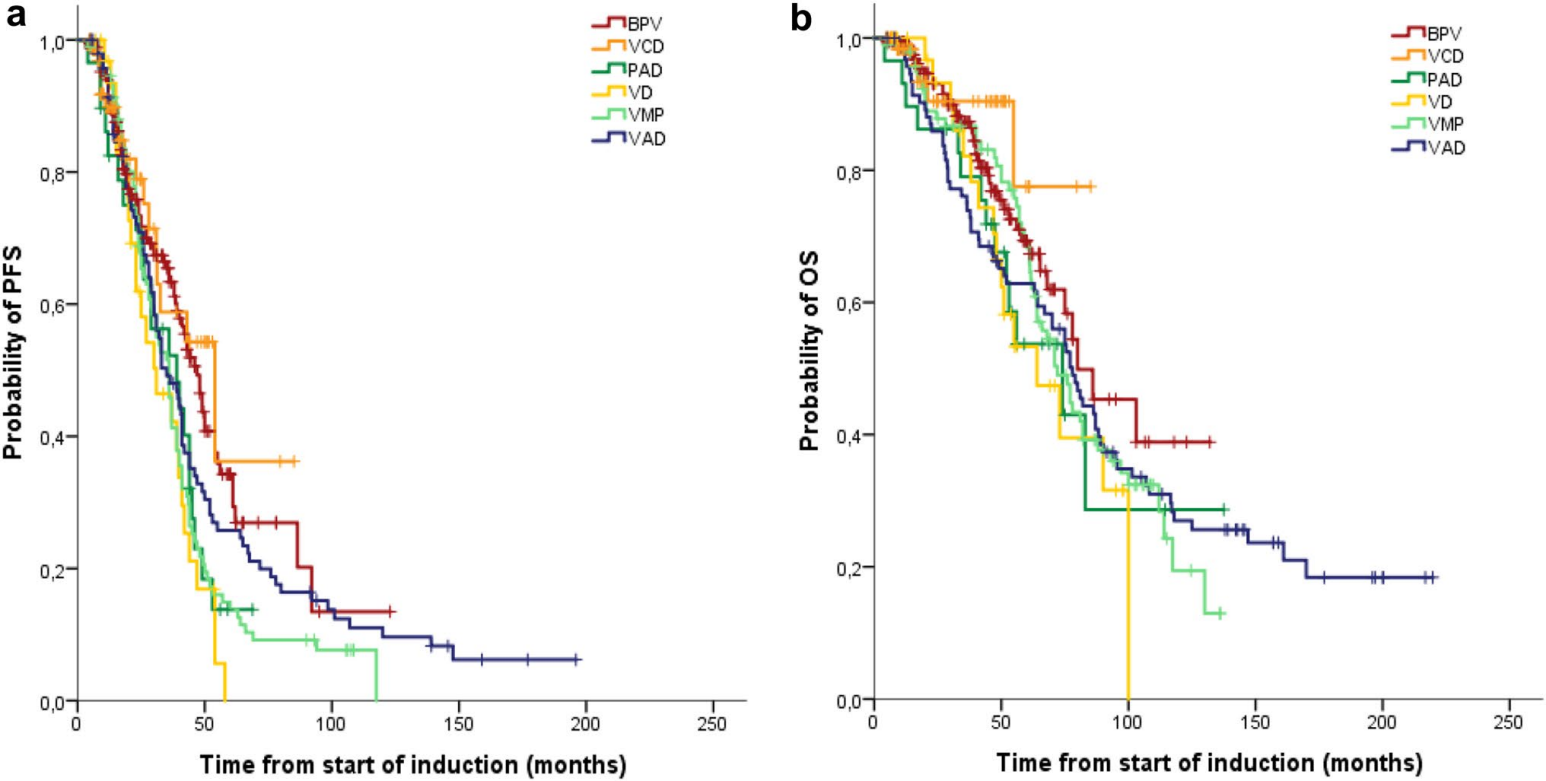
Progression-free survival (PFS) (**a**) and overall survival (OS) (**b**) depending on different induction therapies. An improvement in median PFS was observed in patients treated with the two current induction therapies BPV (47 months) or VCD (54 months) compared to patients treated with the previously used VAD (35 months, *p* < 0.03), VMP (36 months, *p* < 0.01), PAD (39 months, *p* < 0.01) and VD (31 months, *p* < 0.005). The very short follow-up time of only 21 months in the VCD group does not allow a comparison of the OS with the other induction therapies. Median OS was not significantly different between the five other treatment groups, (BPV 80, VAD 78, VMP 72, PAD 74, VD 64 months)

In the last 25 years, we applied a large variety of different induction therapies to reduce tumor burden prior to ASCT (Fig. 3). In the first period up to 2005, induction therapy consisted mainly of conventional chemotherapies, e.g. VAD, VCAP (vincristine, cyclophosphamide, adriblastin, prednisolone) or BP (bendamustine and prednisolone). Following the introduction of the new substances starting in 2006, bortezomib-containing therapies progressively replaced conventional chemotherapies. VMP, which was only approved for non-transplant eligible patients, was temporarily used by us as induction therapy in transplant-eligible patients during the period pending approval of bortezomib-containing induction therapies prior to ASCT. From 2011 onwards, the triple-combination of PAD, VCD and BPV became the most frequent treatment options. Table 2 summarizes the patient characteristics and basic information on therapy for the most commonly used induction regimens. Response rates after induction treatment and first ASCT are shown in Table 3. After completion of induction therapy, the ORR in patients treated with conventional chemotherapy VAD was only 66% with a ≥ VGPR rate of 14% and a ≥ CR rate of 2%. The implementation of various bortezomib-containing regimens significantly improved the ORR to 77–86% (*p* < 0.005), with a ≥ VGPR rate between 29 and 41% (*p* < 0.001) and a ≥ CR rate between 3 and 11% (*p* = 0.09). The majority of BPV-treated patients (*n* = 141; 83%) responded after a median of 2 (range 1–6) 3-weekly induction cycles with 10 sCR (6%), 4 CR (2%), 12 nCR (7%), 40 VGPR (24%) and 75 PR (44%). In contrast, the other induction regimens required longer treatment periods to achieve comparable response rates [e.g. median 3 (range 1–4) 3-weekly PAD cycles (*p* < 0.005) or median 4 (range 2–6) 3-weekly VCD cycles (*p* < 0.001)]. This resulted in a significantly shorter time between initiation of BPV induction therapy and the start of stem cell mobilization e.g. BPV median 71 (range 26–309) days vs PAD median 89 (range 59–183) days (*p* < 0.01) or VCD median 102 (range 45–198) days (*p* < 0.001). There was no significant difference between various induction therapies in the median number of apheresis (1–2) performed and the median yield of CD34^+^ cells (6.5–14.2 × 10^6^ CD34^+^ cells/kg) harvested (Table 2). It was remarkable that a sufficient number of stem cells could also be collected after VMP induction. The median time from the start of induction treatment to ASCT was also significantly shorter in the BPV group at 120 (range 68–431) days compared to VCD 154 (range 98–286) days (*p* < 0.01), PAD 156 (93–328) days (*p* < 0.03), VMP 169 (87–363) days (*p* < 0.001), VD 185 (95–330) days (*p* < 0.001) and VAD 195 (59–517) days (*p* < 0.001). The conditioning therapy prior to ASCT consisted of melphalan 200 mg/m^2^. In patients with concomitant severe renal or pre-existing cardiac impairment, the melphalan dose was reduced to 100 mg/m^2^ (n = 28) or 140 mg/m^2^ (*n* = 68). Autografts contained between 1.1 × 10^6^ and 36.9 × 10^6^ (median 5.2 × 10^6^) CD34^+^ cells/kg. Engraftment was successful in 539 of 540 patients. The ORR after ASCT showed no difference between the patients initially treated with VAD (96%) or with bortezomib-containing therapies (97%). However, the ≥ VGPR (45% vs 76%; *p* < 0.001) and the ≥ CR rate (13% vs 23%; *p* < 0.03) were significantly higher with bortezomib-containing induction regimens. The comparison of the various triple-combinations based on bortezomib showed similar ≥ VGPR and ≥ CR rates (Table 3). An improvement in PFS was observed in patients treated with the two current induction therapies BPV (47 months) or VCD (54 months) compared to patients treated with the previously used VAD (35 months, *p* < 0.03), VMP (36 months, *p* < 0.01), PAD (39 months, *p* < 0.01) and VD (31 months, *p* < 0.005) (Fig. 4a). The very short follow up time of only 21 months in the VCD group does not allow a comparison of the OS with the other induction therapies (Fig. 4b). Among the five other treatment groups, median OS was not significantly different (BPV 80, VAD 78, VMP 72, PAD 74, VD 64 months).

**Table 2:** Patient characteristics depending on the most commonly used induction regimens prior to ASCT and basic information on induction and stem cell mobilization

Parameter	VAD *n* = 95	VMP *n* = 93	BPV *n* = 169	VD *n* = 33	PAD *n* = 29	VCD *n* = 70
Median age, years (range)	57 (38–70)	59 (41–71)	60 (32–75)	60 (40–73)	61 (48–71)	63 (29–72)
eGFR (mL/min)						
≥ 60, *n* (%)	60 (73)	73 (80)	116 (69)	27 (82)	23 (79)	54 (78)
30–59, *n* (%)	18 (22)	16 (18)	23 (14)	3 (9)	4 (15)	12 (17)
15–29, *n* (%)	4 (5)	1 (1)	15 (9)	3 (9)	2 (7)	3 (4)
< 15, *n* (%)	1 (1)	1 (1)	15 (9)	0	0	1 (1)
Unavailable, *n*	12	2	0	0	0	0
Number of cycles median (range)	4 (2–6)	2 (1–6)	2 (1–6)	5 (2–8)	3 (1–4)	4 (2–6)
Time to start mobilization^a^, days, median (range)	156 (59–352)	102 (42–310)	71 (26–309)	121 (56–240)	89 (59–183)	102 (45–198)
CD34^+^cells (× 10^6^/kg), median (range)	12.6 (3.3–70.6)	10.3 (2.5–30.5)	13.5 (1,7–31)	14.2 (2.8–25.2)	6.5 (1–26.7)	11.9 (3.9–67.1)
Number of apheresis, median (range)	1 (1–3)	2 (1–4)	1 (1–4)	2 (1–3)	2 (1–3)	1 (1–3)
Time to ASCT, days, median (range)^b^	195 (59–517)	169 (87–363)	120 (68–431)	185 (95–330)	156 (93–328)	154 (98–286)

VAD: vincristine, adriamycin and dexamethasone; VMP: bortezomib, melphalan and prednisone, BPV: bendamustine, prednisone and bortezomib; VD: bortezomib and dexamethasone; PAD: bortezomb, adriamycin and dexamethasone; VCD: bortezomid, cyclophosphamide and dexamethasone

^a^Time from start induction treatment to stem cell mobilization

^b^Time from start induction treatment to ASCT

**Table 3:** Best confirmed hematological response after completion of induction therapy and three months after the first ASCT

Parameter	VAD *n* = 95	VMP *n* = 93	BPV *n* = 169	VD *n* = 33	PAD *n* = 29	VCD *n* = 70
Response after induction						
sCR, *n* (%)	1 (1)	7 (8)	10 (6)	0	1 (3)	1 (1)
CR, *n* (%)	1 (1)	3 (3)	4 (2)	1 (3)	0	1 (1)
nCR, *n* (%)	0	9 (10)	12 (7)	3 (9)	1 (3)	2 (3)
VGPR, *n* (%)	11 (12)	10 (11)	40 (24)	7 (21)	10 (34)	16 (23)
PR, *n* (%)	50 (53)	43 (46)	75 (44)	17 (51)	13 (45)	39 (56)
* ≥ CR, n (%)*	*2 (2)*	*10 (11)*	*14 (8)*	*1 (3)*	*1 (3)*	*2 (3)*
* ≥ VGPR, n (%)*	*13 (14)*	*29 (31)*	*66 (39)*	*11 (33)*	*12 (41)*	*20 (29)*
*ORR, n (%)*	*64 (66)*	*72 (77)*	*141(83)*	*28 (85)*	*25 (86)*	*59 (84)*
Response after first ASCT						
sCR, *n* (%)	5 (5)	12 (13)	35 (21)	5 (15)	5 (17)	5 (7)
CR, *n* (%)	7 (7)	7 (8)	13 (8)	2 (6)	2 (7)	4 (6)
nCR, *n* (%)	5 (5)	17 (18)	33 (20)	4 (12)	11 (38)	13 (19)
VGPR, *n* (%)	26 (27)	23 (25)	56 (33)	13 (39)	5 (17)	33 (47)
PR, *n* (%)	48 (51)	32 (34)	29 (17)	8 (24)	5 (17)	11 (16)
* ≥ CR, n (%)*	*12 (13)*	*19 (20)*	*48 (28)*	*7 (21)*	*7 (24)*	*9 (13)*
* ≥ VGPR, n (%)*	*43 (45)*	*59 (63)*	*137 (81)*	*24 (73)*	*23 (79)*	*55 (79)*
*ORR, n (%)*	*91 (96)*	*91 (98)*	*166 (98)*	*32 (97)*	*28 (97)*	*66 (94)*
Median PFS (months)	35	36	47	31	39	54
Median OS (months)	78	72	80	64	74	nr^a^

Italic values represent cumulative results

sCR: stringent complete response; CR: complete response; nCR: near-complete response; VGPR: very good partial response; PR: partial response; ORR: overall response rate; nr: not reached

^a^Median observation time in the VCD group is only 21 months

### Role of auto, tandem-auto or auto-RICallo-SCT

**Fig. 5:**
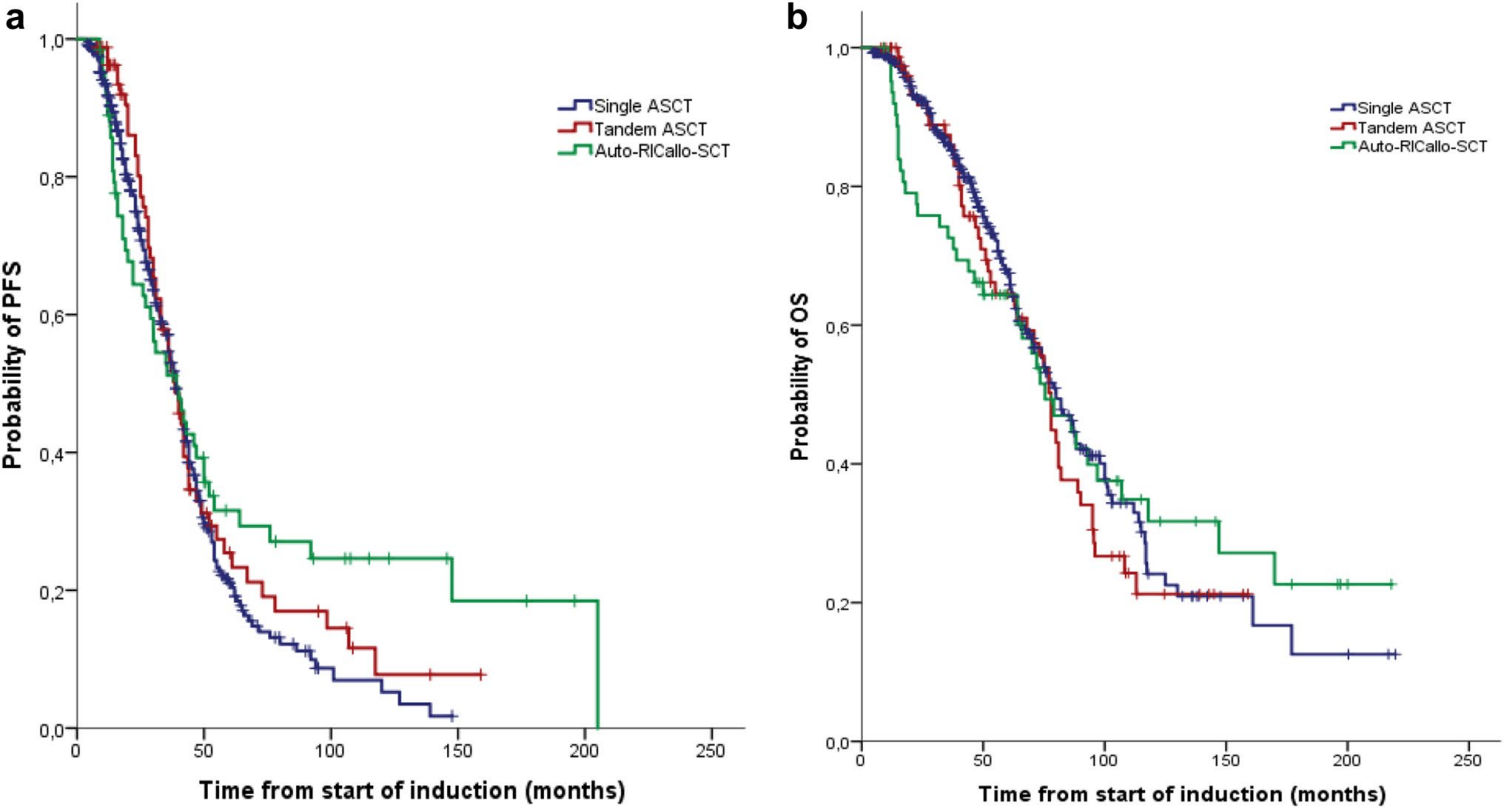
Progression-free survival (PFS) (**a**) and overall survival (OS) (**b**) depending on transplant schedule: auto (*n* = 394), tandem-auto (*n* = 82) or auto-RICallo-SCT (*n* = 64). There was no difference in median PFS between auto-RICallo and single/tandem transplanted patients (39 vs 39 months; *p* = 0.134) and median OS (76 vs 79 months; *p* = 0.964)

Patients received either single ASCT (*n* = 394), or tandem ASCT (*n* = 82) or auto-RICallo SCT (*n* = 64). In particular, patients with partial response to first ASCT were candidates for tandem ASCT or RICallo SCT until 2010. Later, a tandem or RICallo transplant was predominantly performed in patients with high-risk cytogenetics. PFS in patients undergoing single ASCT was 39 and OS 80 months and in the tandem ASCT group, PFS was 39 and OS 78 months. Due to the different transplantation approaches, we refrained from comparing the single to the tandem transplant in our analysis. In 64 patients with an HLA-identical sibling, a RICallo SCT was performed after the first ASCT. The RIC regimen consisted of fludarabine 30 mg/m^2^ for 3 days plus total-body irradiation 2 Gy (Maloney et al. 2003; Björkstrand et al. 2011). There was no difference in median PFS between auto-RICallo and single/tandem transplanted patients (39 vs 39 months; *p* = 0.134) and OS (76 vs 79 months; *p* = 0.964) (Fig. 5a, b). Non-relapse mortality at 24 months was 9% in the auto-RICallo group compared with 2% in the single/tandem auto group (*p* < 0.001). This resulted in a significantly improved 24-month PFS (74 vs 62%; *p* < 0.01) and OS (92 vs 76%; *p* < 0.03) for autologous transplanted patients. However, there was a benefit for auto-RICallo compared to the single/tandem auto patients in the long-term follow-up after 120 months with a PFS of 25 vs 6% (*p* < 0.03) and OS 32 vs 23% (*p* < 0.03), respectively.

### Impact of renal function

**Fig. 6:**
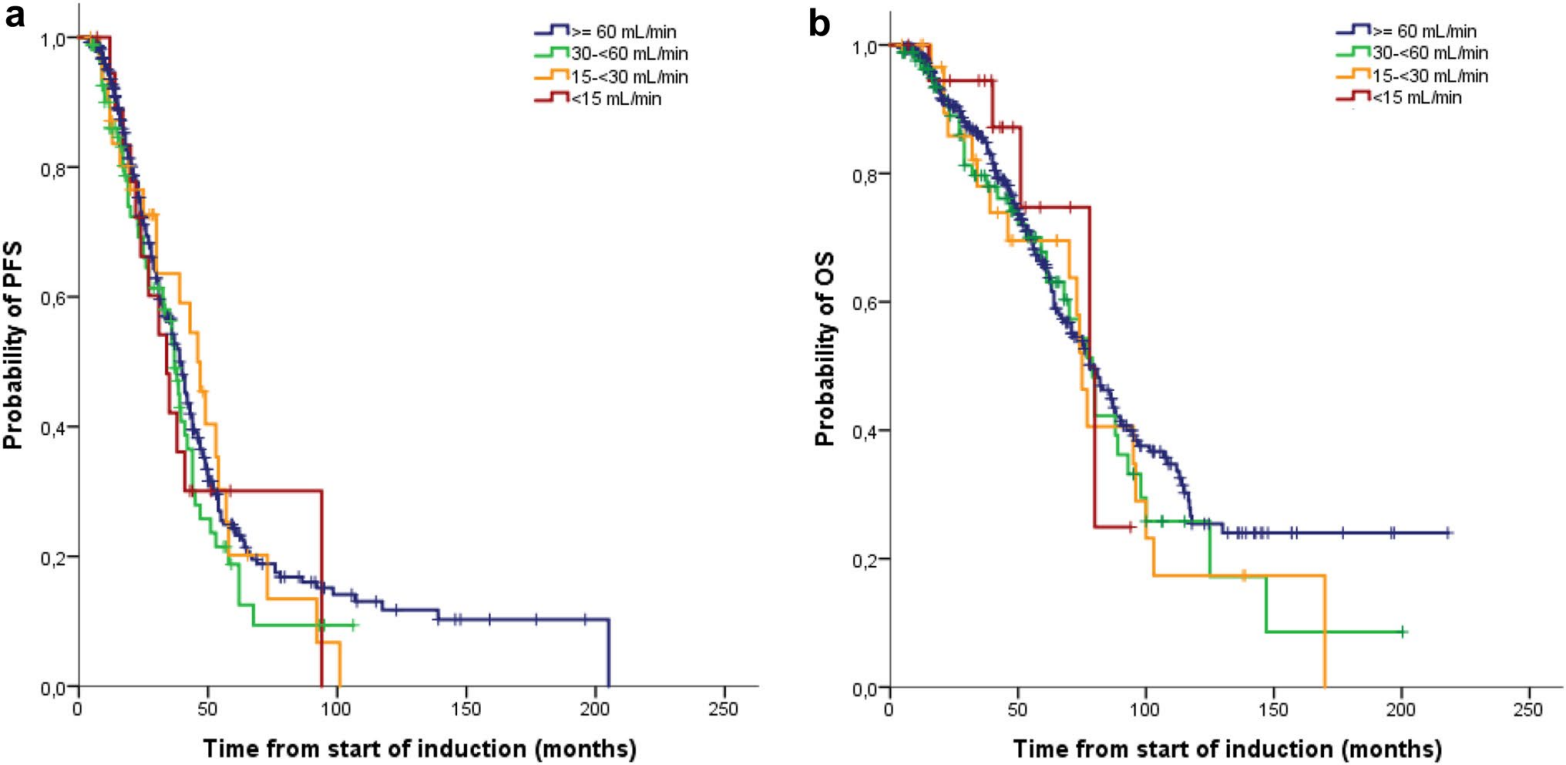
Progression-free survival (PFS) (**a**) and overall survival (OS) (**b**) according to the renal function: eGFR ≥ 60 mL/min (*n* = 382), eGFR 30- < 60 mL/min (*n* = 85), eGFR 15- < 30 mL/min (*n* = 32), eGFR < 15 mL/min (*n* = 19). There was no difference in median PFS between patients with mild, moderate, severe renal dysfunction and renal failure/dialysis (39 vs 37 vs 46 vs 34 months; *p* = 0.58) and in median OS (79 vs 79 vs 75 vs 78 months; *p* = 0.72)

In total 187/518 evaluable patients (36%) had mild renal dysfunction (eGFR: 60–89 mL/min), 85 (16%) moderate renal dysfunction (eGFR: 30–59 mL/min), 32 (6%) severe renal dysfunction (eGFR: 15–29 mL/min) and 19 (4%) renal failure/dialysis (eGFR < 15 mL/min). The majority of patients with severe renal dysfunction/ renal failure/dialysis (*n* = 30; 59%) received induction treatment with BPV. We observed no difference in median PFS between patients with mild, moderate, severe renal dysfunction and renal failure/dialysis: 39 vs 37 vs 46 vs 34 months (*p* = 0.58), and in median overall survival: 79 vs 79 vs 75 vs 78 months (*p* = 0.72) (Fig. 6a, b). Forty-one of the 51 patients with eGFR < 30 mL/min (80%) improved their renal function after the first ASCT. Seventeen (33%) patients reached CRrenal, 5 (10%) patients PRrenal and 19 (37%) patients MRrenal. Seven of the 11 dialysis-dependent patients became dialysis-independent.

## Discussion

In this retrospective study, we present the results of a large, single-center-cohort of 540 unselected NDMM patients treated with ASCT. In our analysis, the median PFS of 39 months and OS of 79 months are comparable to those observed in other ASCT studies conducted in the last 30 years (Fermand et al. 1998; Harousseau et al. 2010; Sonneveld et al. 2012). In addition to the 401 patients who fulfill the restrictive criteria for inclusion in most clinical phase 2/3 studies, our analysis included 139 patients with at least one clinically relevant comorbidity, which would usually have led to study exclusion. In this subgroup of MM patients who did not fulfill the inclusion criteria for clinical trials, but who were considered as transplant eligible by us, PFS was shortened only slightly and OS was not reduced. This is concordant with the results of the Danish MM registry, which also found no difference in OS between these two groups (Klausen et al. 2019). This suggests that in clinical practice, significantly more patients could benefit from an ASCT than are usually included in transplant studies.

During the last 20 years, there has been a complete shift in pre-transplant induction therapies from conventional chemotherapy to combination therapies using novel agents (Nooka et al. 2013; Kumar et al. 2014). This change in induction therapy is mirrored in our retrospective analysis. While VAD (75%) was the predominant conventional chemotherapy until 2005, a transition phase up to 2010 was followed by a complete switch to bortezomib-containing combinations. The remission rates after ASCT achieved in our first cohort up to 2005 with a CR rate of 11% and a ≥ VGPR rate of 39% are comparable to the CR rates between 6 and 44% and ≥ VGPR rates between 36 and 57% in other transplant studies using conventional chemotherapy induction regimens (Koreth et al. 2007; Sonneveld et al. 2012; Nooka et al. 2013). However, our observed PFS of 39 months and OS of 80 months after induction therapy with VAD was considerably longer than in most studies, comparable PFS of 24 and 42 months and OS of 65 and 82 months being achieved only with additional consolidation or maintenance therapy (Fermand et al. 1998; Bladé et al. 2005; Goldschmidt et al. 2018). The reason for our favorable results in the first cohort could be the high proportion of auto-RICallo transplanted patients (35%), our early extensive implementation of thalidomide monotherapy starting in 2000 and our use of triple combinations including thalidomide (Pönisch et al. 2008) and bortezomib since 2004 (Pönisch et al. 2013) in a relapse setting. After the introduction of bortezomib-containing triple combinations (VMP, BPV, PAD and VCD) in induction therapy, there was a significant improvement in response rates after induction. In comparing these different triple therapies, we found similar ORR between 77 and 86% and ≥ VGPR rates between 29 and 41%. The median duration of BPV induction to achieve best response was only 6 weeks and thus significantly shorter than the 9–12 weeks required for other bortezomib-containing triple combinations. This shorter time to best response is clinically relevant because rapid tumor control is usually associated with a corresponding improvement in clinical symptoms and a lower risk of bortezomib-associated polyneuropathy. The mobilization and collection of stem cells were feasible and effective among the various triple combinations, comparable to the published data for PAD, VCD (Mai et al. 2015) and BPV (Poenisch et al. 2015). It was remarkable, however, that a sufficient number of stem cells could also be collected after VMP induction. The reason for this could be the low cumulative dose of oral melphalan (median 72 mg/m^2^) in the VMP induction, resulting in limited stem cell toxicity. Following the first ASCT, the ORR of the different bortezomib-containing triple combinations increased to 94–98% with a ≥ VGPR rate between 63 and 81% and CR rate between 13 and 28%. These response rates compare favorably with those reported by Moreau et al. (2011); Sonneveld et al. (2012); Moreau et al. (2019) and Goldschmidt et al. (2020) for alternative bortezomib-based regimens PAD, VCD and VTD. Both the BPV and VCD induction therapies, which we have used preferentially since 2011, are associated with a significantly better PFS compared to the other triplets, although BPV had only a slightly (but not statistically significant) better OS compared to the triplets VMP and PAD. Compared to the bortezomib-containing triple combinations, the doublet bortezomib and dexamethasone showed significantly shorter PFS and OS despite the significantly longer duration of induction therapy. Therefore, our results indicate a higher efficacy of triplet compared to doublet therapies. Our retrospective analysis found no difference in median PFS and OS between patients transplanted with single and tandem ASCT, or auto-RICallo SCT. The auto-RICallo transplant patients showed significantly better long-term survival. However, a significantly increased early mortality needs to be expected in the first few years. This is in line with the results of the EBMT-NMAM2000 study, which compared auto-RICallo SCT with tandem ASCT in a prospective phase 3 trial (Björkstrand et al. 2011). The majority of patients with severe renal dysfunction or renal failure/dialysis received an induction treatment with BPV. As induction therapy, this combination has shown high efficacy and good tolerability in both transplant-eligible and non-transplant-eligible MM patients with renal impairment (Pönisch et al. 2014; Poenisch et al. 2015; Holzhey et al. 2021). Specifically, this combination induced a rapid reduction in monoclonal LC production in the first few days of treatment, potentially preventing the development of irreversible renal failure (Pönisch et al. 2015; Tessenow et al. 2017; Holzhey et al. 2021). The German-Speaking Myeloma Multicenter Group (GMMG) previously reported that the bortezomib-based triplet therapy PAD before ASCT could overcome the negative prognostic impact of renal impairment (Scheid et al. 2014). Our results confirm this for the bortezomib-containing inductions used predominantly here, as we found no differences in PFS and OS in patients with severe renal impairment compared to patients with normal or moderate restricted renal function.

In conclusion, our real-world data demonstrate the substantial value of ASCT as a first-line treatment for younger MM patients. In addition to patients meeting restrictive inclusion and exclusion criteria for clinical studies, patients with relevant comorbidities (e.g. severe renal impairment) classified as eligible for transplantation also benefit from an ASCT. The risk profile of patients transplanted in our clinic has changed substantially over the past 25 years: median age increased from 57 to 62 years and significantly more patients with reduced general condition (ECOG ≥ 2) were transplanted. While conventional chemotherapy was administered until 2005 after a transitional phase from 2011, exclusively bortezomib-based combinations became established as induction therapy prior to ASCT. The different bortezomib-containing triplets resulted in similar ORR and OS. Only improvement in PFS was observed in patients treated with the two current induction therapies BPV and VCD in comparison with the previously used VMP, PAD and the doublet VD. In addition, the significantly shorter duration of BPV induction therapy indicates a superior efficacy of this combination compared to the other bortezomib-based inductions.
